# Biophysical Control of Bile Duct Epithelial Morphogenesis in Natural and Synthetic Scaffolds

**DOI:** 10.3389/fbioe.2019.00417

**Published:** 2019-12-13

**Authors:** Anette Funfak, Latifa Bouzhir, Emilie Gontran, Nicolas Minier, Pascale Dupuis-Williams, Samy Gobaa

**Affiliations:** ^1^Institut Pasteur, Biomaterials and Microfluidics Core Facility, Paris, France; ^2^Université Paris-Saclay, UMR-S1174 INSERM, Orsay, France; ^3^Université de Technologie de Compiègne, Alliance Sorbonne Université, Compiègne, France; ^4^ESPCI, PSL University, Paris, France

**Keywords:** bile duct, microenvironment, organoids, hydrogels, 3D cell culture

## Abstract

The integration of bile duct epithelial cells (cholangiocytes) in artificial liver culture systems is important in order to generate more physiologically relevant liver models. Understanding the role of the cellular microenvironment on differentiation, physiology, and organogenesis of cholangiocytes into functional biliary tubes is essential for the development of new liver therapies, notably in the field of cholangiophaties. In this study, we investigated the role of natural or synthetic scaffolds on cholangiocytes cyst growth, lumen formation and polarization. We demonstrated that cholangiocyte cyst formation efficiency can be similar between natural and synthetic matrices provided that the mechanical properties of the hydrogels are matched. When using synthetic matrices, we also tried to understand the impact of elasticity, matrix metalloprotease-mediated degradation and integrin ligand density on cyst morphogenesis. We demonstrated that hydrogel stiffness regulates cyst formation. We found that controlling integrin ligand density was key in the establishment of large polarized cysts of cholangiocytes. The mechanism of lumen formation was found to rely on cell self-organization and proliferation. The formed cholangiocyte organoids showed a good MDR1 (multi drug resistance protein) transport activity. Our study highlights the advantages of fully synthetic scaffold as a tool to develop bile duct models.

## Introduction

Cellular microenvironment cues, including surface topography, substrate rigidity and biochemical signals determine to a large extent the outcome of many biological processes (Tan et al., 2003; Gobaa et al., 2011; Unadkat et al., 2011). Strikingly, the exposure of stem cells to mechanical cues allows steering the differentiation process (Engler et al., 2006). Gaining control over the mechanical stress landscape of the cell was cornerstone in the development of successful organ-on-chip and strategies (Huh et al., 2013; Benam et al., 2016) and of advanced 3D culture systems (Gjorevski et al., 2016).

Recapitulating a physiological hepatic function *in vitro* is currently a very active line of research. Approaches, based on the use of primary adult hepatic cells (Broutier et al., 2016) or on the controlled differentiation of induced Pluripotent Stem Cells (iPSC) (Takebe et al., 2014) have led to the derivation of new organotypic models. These advanced cell culture systems were developed in order to improve on the physiological relevance of standard 2D culture hepatic cell lines. Despite great successes (Broutier et al., 2016) in recapitulating the hepatic function *in vitro*, the inclusion of a biliary tree capable of removing the toxic metabolite is yet to be developed. The biliary tree is composed of intra and extra hepatic ducts organized in a complex network of interconnected tubes with luminal diameters ranging from <15 μm to a few mm capable of carrying the bile from the liver to the intestine (Roskams et al., 2004; Boyer, 2013). The bile ducts are lined with the epithelial cholangiocytes which regulate, through secretion and adsorption, the composition and flow of the bile (Bogert and LaRusso, 2007).

The biliary system is itself the target of a vast array of liver pathologies called cholangiopathies (Cheung et al., 2018) that account for a large proportion of liver transplants (Murray et al., 2005). Today's treatments of cholangiopathies consist mostly in either largely ineffective pharmacotherapies or in surgeries that are restricted to the extrahepatic large duct. Living donors of liver grafts often suffer from biliary complications like stricture formation, bile leaks and infection (De Assuncao et al., 2017).

The field of bile duct engineering, previously hindered by the lack of available cells, has recently benefited from progress in the understanding of biliary physiopathology and the availability of cholangiocytes cell lines or methods recapitulating differentiation of hepatic precursor cells, stem cells or iPSCs, using various mixtures of growth factors known to direct bile duct embryogenesis (Dianat et al., 2014; De Assuncao et al., 2015; Ogawa et al., 2015; Sampaziotis et al., 2015; Takayama et al., 2016). What remains to be explored when considering developing innovative liver therapies is to understand how the microenvironment guides the self-organization of cholangiocytes into functional biliary tubes (Shiojiri and Sugiyama, 2004; Raynaud et al., 2011).

Cell culture in 3D has shown that cholangiocytes possess *in vitro* morphogenetic capabilities in natural hydrogels like collagen or Matrigel (Ishida et al., 2001; Tanimizu et al., 2007; Hashimoto et al., 2008; Kido et al., 2015). The scaffolding properties of Matrigel help supporting the 3D reorganization of adult cells. The production of “cholangioids” in Matrigel from healthy cholangiocytes versus issued from patients with primary sclerosing cholangitis (PSC) (Soroka et al., 2019) has recently exemplified their interest for the study of pathogenicity of biliary diseases and their putative use in the identification of therapeutic targets (Loarca et al., 2017). Matrigel embedding is also used as a final step in most cholangiocytes differentiation protocols, to assess their capacity to organize in biliary cysts (Dianat et al., 2014; De Assuncao et al., 2015; Ogawa et al., 2015; Sampaziotis et al., 2015; Takayama et al., 2016). However, relaying on hydrogels based on natural extracellular matrix (ECM) clearly hinders the mechanistic understanding of organogenesis or cysts formation as these scaffolds prohibits the decoupling of the biophysical and biochemical signaling. Similarly, liver stiffness is a key marker of liver pathology (Wells, 2008; Mueller and Sandrin, 2010) and marks the development of chronic fibrotic diseases (Saneyasu et al., 2016). In this context using liver organoids to study disease progression begs for the development of hydrogel systems with tunable mechanical properties. Interestingly, the stiffness-dependent differentiation of cholangiocytes seems to be modulated by the biochemical nature of the used matrix (Kourouklis et al., 2016). This indicates the high level of intricacy of both the biochemical and biophysical cues for these finely regulated biological processes.

This complex, and sometime undefined, nature of ECM-based hydrogels including Matrigel (MT) hinders the mechanistic understanding of organogenesis in a dish. On the other hand, synthetic hydrogels technology, such as systems based on the polymerization of polyethylene glycol (PEG), underwent many developments in the last decades placing them at the top of the list of the most physiologically relevant yet fully defined substrates for cell culture (Gjorevski et al., 2016). Today, synthetic hydrogel systems gained key functionalities including physiological elastic moduli (Ranga et al., 2014), easy tethering of biochemical ligands (Mosiewicz et al., 2013) and matrix metalloprotease (MMP) mediated proteolytic degradation allowing cell migration (Lutolf and Hubbell, 2003). These engineered functionalities opened the door for the establishment of advanced and physiologically relevant assays in fully synthetic scaffolds including the production of intestinal organoids (Gjorevski et al., 2016), the differentiation of embryoid bodies into neural tubes (Ranga et al., 2016) and the production of epithelial cysts (Enemchukwu et al., 2016). Therefore, the side-by-side comparison of organotypic development in both synthetic (PEG) and natural (MT) hydrogels could help with defining the set of microenvironment features that are essential to the proper self-organization of the seeded cells.

Cholangiocytes, like most other epithelial cells are able, when embedded in natural ECM like Matrigel or collagen, to self-organize into polarized monolayers enclosing a central lumen termed cyst. These structures are good models for studying duct morphogenesis since they share with the tubes the same topology and the same organization of the epithelium, where the polarized cells are connected by adherens and tight junctions, thus ensuring secretion, cohesiveness, and coordinated growth of the monolayer. Here we report on the quantitative impacts of biochemical and biophysical cues on the development of cholangiocyte cysts *in vitro*. We demonstrate that cholangiocytes can organize and produce polarized cysts in both natural and synthetic scaffolds. We also show that the biophysical and biochemical parameters of the extracellular milieu are key for cyst morphogenesis. We also report on the dynamics of lumen formation and how this process is influenced by the extracellular milieu.

## Materials and Methods

### Cell Culture

Normal Rat Cholangiocytes (NRC) cell line was obtained from N. LaRusso's laboratory (Vroman and LaRusso, 1996). The cells were cultured on collagen coated T25 flasks and maintained in DMEM F12 medium supplemented with 5% fetal bovine serum (Life Technologies), 1× Antibiotic-Antimycotic (Life Technologies) and growth active factors including Insulin (Life Technologies), Dexomethosane (Sigma), 3,3,5-Triiodo-L-thyroninen sodium salt (Sigma), Bovine Pituitary Extract (BPE, Life Technologies) were added as described in de Groen et al. (1998).

### Hydrogel Formation

Synthetic hydrogels were formed by Michael-type addition of bicystein-bearing peptides onto star-shaped, vinylsulfone-functionalized, polyethylene glycol macromeres. Lyophilized peptides were ordered from Pepmic (China). Peptide sequences 16A (Ac-GCRD-GPQG↓I**A**GQ-DRCG-NH2) and 16R (Ac-GCRD-VPMS↓M**R**GG-DRCG-NH2) were chosen in order to ensure varying MMP sensitivity as established elsewhere (Lutolf and Hubbell, 2003; Patterson and Hubbell, 2010). An RGD peptide (Ac-GCGYG**RGD**SPG-NH2) was used at various concentrations in order to promote the integrin mediated adhesion of encapsulated cells. Briefly, peptides were resuspended in Milli-Q water to obtain a 12% (w/v) working solution. Before using these peptides to form hydrogel, we controlled their integrity and purity on a Bruker UltrafeXtreme MALDI-TOF/TOF instrumentation (Bruker-Daltonics, Germany). One microliter of peptide at 0.1 mg/ml was deposited on a MTP 384 ground steel target plate with 1 μl of 2,5-Dihydroxybenzoic acid (2,5-DHB) in 50% acetonitrile, 0.1% trifluoroacetic acid as matrix solution. Data were acquired using Flexcontrol software (Bruker-Daltonics, Germany) and shots were recorded in positive ion reflectron mode. Mass spectra were externally calibrated in the m/z range of 700–3,500 Da with a peptide calibration standard (Bruker-Daltonics, Germany) and analyzed with the Flexanalysis software (Bruker). Next, we performed an Ellman assay as per the manufacturer instructions in order to quantify free thiol concentration in each peptide working solution. The difference between theoretical and measured thiol concentration was taken in consideration in order to produce hydrogels with the desired Thiol (Th)/Vinyl Sulfone (VS) stoichiometry. Finally, we measured the peptide net content by hydrolyzing the samples in 1% phenol 6 N HCL for 20 and 48 h at 110°C in presence of known amount of NorLeucine as internal standard. After HCL evaporation the samples are analyzed on a L-8800 Hitachi amino acids analyzer (post-column derivatization with ninhydrin after ion-exchange chromatography separation).

Eight-arm polyethylene glycol-vinyl sulfone or PEG-VS 40 kDa macromeres were ordered as a custom synthesis from NOF (Japan). Lyophilized powder was dissolved in a 0.3 M, pH 7.5, HEPES buffer (Dominique Dutscher, France) at 12% (w/v). Hydrogel sterility was ensured by filtering both peptide and PEG-VS working solutions on a 0.22 μm syringe filter (Millex-GS, Merck). Polyethylene glycol (PEG) hydrogels were produced as described in Lutolf and Hubbell (2003). Rapidly, 4.5% (w/v) containing 100 μM RGD gels were formed by mixing 63 μl PEG-VS macromere (12% w/v) with 103 μl HEPES buffer, 2 μl RGD peptide (10 mM) and 40 μl NRC cells resuspended in HEPES buffer with a cell concentration of 1.5 × 10^6^ cells/ml. In a second step we added 12 μl of 16R or 16A peptide working solutions (12% w/v). Before gelation, the mixture was pipetted up and down gently. Fifty microliters drops of gel mixture were sandwiched between two mirror-polished PTFE pieces separated with a 1 mm spacer in order to produce 50 μl hydrogel disks. The assemblage was then placed in a Heracell 150i incubator (ThermoFisher Scientific, Waltham, MA USA) at 37°C, 100% relative humidity for 30 min. Upon crosslinking, gel disks were demolded and transferred to a 12 well plate (Falcon® multiwell plate, Corning) loaded with the adequate cell culture medium.

For Matrigel experiments, NRC cells were first mixed with a 7 mg/ml growth factor reduced Matrigel (Corning) solution and then diluted to 3.5 mg/ml with pre-cooled NRC complete medium on ice with a cell concentration of 3 × 10^5^ cells/ml. Before the filling of 8-well μ-slides with the Matrigel-cell mixture, the pre-cooled slides were coated with 50 μl 3.5 mg/ml Matrigel solution and polymerized for 15 min at 37°C and 5% CO_2_. Subsequently 300 μl of Matrigel-cell mixture was pipetted in each well and polymerized for 15 min at 37°C and 5% CO_2_ before adding cell culture medium.

For both hydrogel systems NRC cells were pre-dissociated by up and down pipetting of the cell suspension followed by a post-dissociation step using a 40 μm cell strainer (Merck). The dissociated cell suspension was then diluted to the aimed cell concentration and mixed with the hydrogel solutions. It is to mention, that the natural and synthetic hydrogel system required a different state of cell dissociation (low for PEG hydrogel and high for Matrigel) due to the system requirements and the inhibition of NRC cell migration in PEG hydrogels. This was achieved by the adjustment of the pre-dissociation step.

### Rheology

The hydrogel shear modulus was obtained by rheometric measurement with a Kinexus Ultra Plus Rheometer (Malvern Panalytical, UK). Briefly, gels of different concentration were casted as cylinders of 4 mm in diameter after swelling in water overnight. The rheometer was fitted with a 4 mm geometry. Each gel disk was compressed to 80% of its nominal height in order to avoid slippage during the measurements. An amplitude sweep was performed to determine the linear viscoelasticity region (LVER) of the measured material. Shear moduli were extracted from a frequency sweep analysis performed with a constant 1% strain. The reported G′ values were obtained at 0.5 Hz.

The hydrogel shear modulus for Matrigel was obtained by a direct polymerization of the polymer solution on the working stage of the rheometer. Therefore, samples of 314 μl were added to the center of the pre-cooled (4°C) lower working plate. The geometry of 20 mm was then immediately lowered before the gel started to form, to a working gap of 1 mm. For the polymerization of the polymer suspension, the temperature was increased stepwise with a rate of 5°C/min from 4 to 37°C and kept at 37°C during measurement. A solvent trap cover was used to prevent evaporation effects around the working stage. Rheometric measurements were performed at 0.5 Hz and a constant 0.1% strain.

### Viability/Cytotoxicity Assay

PEG and Matrigel hydrogels were washed with PBS and then incubated for 30 min at 37°C, 5% CO_2_ with 4 μM Ethidium homodimer-1 and 2 μM Calcein, AM (ThermoFisher scientific) in growth medium. After a second washing step, the gels were imaged as described below. In average 12 images were acquired per condition. Fluorescence intensity was measured after background correction and normalized for the variations in cyst surface using ImageJ, an image analysis software available on the NCBI website (https://imagej.nih.gov/ij/).

### Functionality Assays

To demonstrate the multidrug resistance protein 1 (MDR1) transporter activity of single or multi-lumen cysts, polyethylene glycol (PEG) and Matrigel hydrogels were treated with 50 μM rhodamine 123 (Rh123, Sigma) in serum free medium for 1 h at 37°C. After a washing step (3× in serum free medium) the hydrogels were incubated for 2 h at 37°C, 5% CO_2_ in fresh complete medium. To inhibit the MDR1 transporter activity the cysts containing hydrogels were incubated with 50 μM verapamil (Sigma-Aldrich) at 37°C for 30 min before adding Rh123. The rhodamine assay was then repeated as described above. For the quantification of the Rh123 assay the number of cysts containing Rh 123 in the luminal space and the number of cysts showing Rh 123 signal only at the level of the epithelium (blocked cysts) were counted with ImageJ.

### Immunostaining of the Cysts

Immunostaining were performed without extracting the NRC cysts from PEG hydrogel or Matrigel. The PEG hydrogels were washed with PBS and then fixed with 4% paraformaldehyde (PFA) overnight at 4°C. Cysts were permeabilized with 0.5% Triton X-100 in PBS for 1–2 h at 4°C and then blocked with 2% BSA, 0.1% Tween-20 in PBS for 3 h. The primary polyclonal anti-E-cadherin antibody (Thermo Fisher Scientific PA5-32178, 1: 400) was diluted in blocking solution and incubated for 2–4 days at 4°C. Unbound primary antibodies were washed by incubating the gels in blocking buffer (3 times, 10 min each). The secondary antibody Goat anti-Rabbit IgG (H+L), Alexa Fluor Plus 647 (Thermo Fisher Scientific A32733, 1:400) was diluted in blocking solution and incubated for 2–4 days at 4°C. Nuclei and f-actin filaments were stained by adding 10 μM Hoechst and 16.5 nM Phalloidin 488 (Thermo Fisher Scientific) in PBS containing 1% BSA. Gels were incubated for 2 days at 4°C. Stained gels were then washed intensively in PBS.

Matrigel hydrogels were washed with PBS and fixed with 4% paraformaldehyde and 5% sucrose in PBS. Cyst were permeabilized with 0.5% Triton X-100 in PBS for 30 min and then blocked with 0.1% BSA and 1% goat serum for 30 min at RT. The primary polyclonal anti-E-cadherin antibody (Thermo Fisher Scientific PA5-32178, 1: 400) and 16.5 nM Phalloidin 488 (Thermo Fisher Scientific) were diluted in blocking solution and incubated overnight at 4°C. The Matrigel gels were washed by incubation in PBS containing 0.05% Tween (3 times, 10 min each). The secondary antibody Goat anti-Rabbit IgG (H+L), Alexa Fluor Plus 647 (Thermo Fisher Scientific A32733, 1:400) was diluted in blocking solution and incubated for 1 h at RT. After washing, the gels were mounted with Prolong Gold antifade reagent (Invitrogen Molecular probes) containing DAPI for nuclei staining.

### Imaging

Wide field microscopy was performed on an inverted axio Observer Z1 inverted microscope from Zeiss (Carl Zeiss Microscopy GmbH, Jena, Germany) equipped with an OrcaFlash 4 V2.0 camera (Hamamatsu, Hamamatsu city, Japan) and Plan-Apochromat phase 1 10× (NA, 0.45) or Plan-Apochromat phase 2 20× (NA, 0.5) objectives. In case of live imaging (cyst growth in PEG hydrogel) temperature and CO_2_ were ensured with a PeCon incubator equipped with the corresponding CO_2_ and temperature modules.

Confocal imaging was performed on an SP8 inverted microscope (Leica Microsystems, Germany) equipped with HC FLUOTAR L 25×/0.95 W 0.17 VISIR objective and a white laser system. Confocal imaging of cyst polarization in Matrigel was acquired with a confocal microscope Nikon Eclipse TE-2000-E equipped with a X20/0.45 Plan Fluor ELWD Ph1 DM objective (∞/0–2 WD 7.4).

### Quantification of Cysts Growth and Morphology

For the quantification of cyst growth, cyst and lumen formation in PEG and Matrigel hydrogels up to 15 z-stacks per condition and time point were acquired. Between 2 and 3 z-stacks were taken from non-overlapping regions within a hydrogel. Z-stacks were performed from the bottom to the top of the hydrogel with fixed height and image sampling number for all condition. Cyst size, number of cysts and number of single- or multi-lumen formed per imaged z-stack was then analyzed using Matlab Image processing toolbox. The cyst formation frequency was determined by relating the cyst number per z-stack to the corresponding initial cell number per z-stack. Lumen formation was also characterized by staining for actin, E-cadherin, and DNA as described above.

### Statistical Analysis

All values are expressed as mean±SEM except for the rheological and viability measurements (mean±SD). Statistical significance was determined using Kruskal-Wallis *t*-test. The dynamics of cyst formation and single lumen production were analyzed in R (3.6.0) or the R project for statistical computing downloadable from https://www.r-project.org/. The fitted curves were generated with the GLM function fed with the models: Cyst frequency ~ scaffold * log(Day) and single lumen ~ scaffold * exp(Day). The obtained *p*-values were corrected for multiple comparisons with the Benjamini and Yekutieli method (BY) (Benjamini and Yekutieli, 2001).

## Results

### Normal Rat Cholangiocytes Form Cysts Upon Culture in 3D Scaffolds

**Figure 1:**
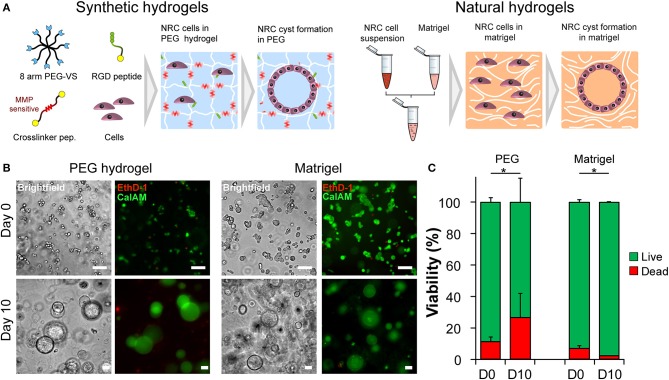
Normal Rat Cholangiocyte (NRC) encapsulation and viability assessment. **(A)** Schematic representation of the used encapsulation procedure showing the differences between PEG (synthetic) and Matrigel (natural) hydrogels in terms of composition. In order to assess the self-organization capabilities of NRCs in synthetic and natural matrices, harvested cells were seeded into PEG hydrogels and Matrigel. Eight-arm PEG macromers bearing a vinyl sulfone function at the end of each arm were crosslinked with different peptides sensitive to matrix metalloprotease degradation in order to produce synthetic hydrogels. These synthetic PEG scaffolds were further functionalized with integrin ligands and proteolytic degradation capabilities in order to ensure NRC survival whereas in Matrigel encapsulated NRCs naturally formed cysts. **(B)** Brightfield and fluorescent images of live/dead staining of NRCs cultured in PEG and MT for 3 h or 10 days. The NRC cells when maintained for 10 days in culture formed hollow 3D structures, termed cysts. **(C)** When viability was assessed we found that the obtained structures were viable with PEG hydrogel causing slightly higher cell mortality. Represented PEG hydrogels condition corresponds to 4.5% (w/v) functionalized with 1,200 μM RGD ligand. *n* = 3. *Significant at *p* < 0.05 in a Kruskal-Wallis test. Error bars represent SD. Scale bar equal 100 μm.

Triggering morphogenetic processes and achieving high levels cell-self organization is best recapitulated when initiated in the presence of a three-dimensional support or scaffold. The initial objective of this study was to identify 3D microenvironments supportive of cholangiocytes self-organization. Based on observations made with other epithelial cell lines we first seeded Normal Rat Cholangiocytes (NRC) in both synthetic, fully defined polyethylene glycol (PEG) hydrogels and in natural extracellular matrix-based Matrigel (MT) (Figure 1A). We quickly confirmed that NRCs can survive and proliferate in both conditions. Over a culture period of 10 days, cultured NRCs self-organized into hollow spheres or cysts where a very thin layer of epithelial cells marked the boundary between an inner lumen and the extracellular milieu (Video S1). We also observed that unlike in MT, NRCs cannot migrate in PEG and that cyst formation was mostly due to the seeding of small cell aggregates rather than single cells. When assessing cell viability, we found that encapsulated NRC showed globally a good viability in PEG and in MT. At day 10 a modest decrease in viability was observed in PEG, most probably due to the death of the cells that could not form cysts (Figures 1B,C). Overall, the growth kinetics (Video S2) in both materials allowed for the formation of cysts with sizes ranging from 40 to 800 μm. At this stage, we could not observe obvious differences between cysts cultured in either synthetic or natural scaffolds.

### Matrix Stiffness and Integrin Ligand Density Modulate Cyst Formation

**Figure 2:**
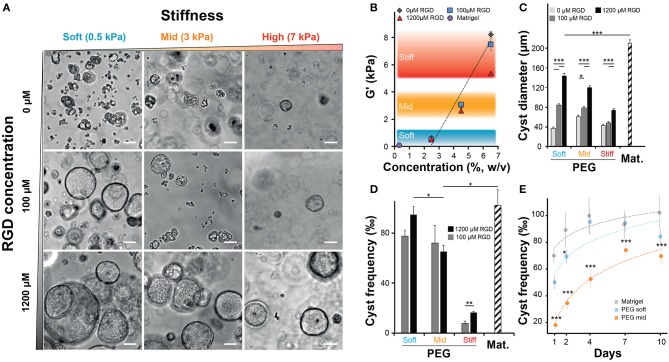
Impact of matrix stiffness and integrin ligand density on Normal Rat Cholangiocytes (NRC) cyst formation. **(A)** Brightfield images of NRC cysts cultured for 10 days. NRCs were encapsulated in PEG hydrogels crosslinked with an MMP sensitive peptide (16R) and varying for concentration (w/v) and integrin ligand density. **(B)** Quantification of the impact of varying PEG and RGD concentration on the elastic modulus or G′ indicating the stiffness of produced PEG hydrogels. There we defined three stiffness domains including soft (2.5% PEG w/v, around 0.5 kPa), intermediate (4.5% PEG w/v, around 3 kPa) and stiff gels (6.5% PEG w/v, >5.5 kPa). The addition of up to 1,200 μM RGD had an overall moderate impact on the elasticity regime. **(C,D)** Quantification of cyst size and cyst frequency, respectively, at day 10 across soft, intermediate, and stiff polyethylene glycol gels and in Matrigel. **(E)** Dynamic study showing the evolution of the frequency of cyst production across the studied conditions. *, **, and *** Significant at *p* < 0.05, *p* < 0.01, and *p* < 0.001, respectively. *n* = 4. Error bars represent SEM. Scale bar equal 100 μm.

After confirming that NRC could self-organize into 3D structures reminiscent of polarized cysts. Unlike unpolarized cell aggregates, the obtained structure clearly showed a large lumen, cells organized as a monolayer that are staining positive for actin on the apical side and for E-cadherin on the basolateral side. First, we sought to investigate the determinants of cyst formation dynamics across gel composition (natural vs. synthetic matrices), matrix stiffness (or elastic modulus noted G′) regime and integrin ligand density. Initially, we varied both the PEG concentration and the integrin ligand density (Figure 2A) of the synthetic hydrogel system while documenting the impact of PEG concentration of the elastic modulus (Figure 2B). Unlike ECM-based scaffolds, PEG hydrogels can be produced at varying elastic moduli while maintaining a constant molarity of integrin ligand (Gobaa et al., 2011). In this scenario the impact of increased RGD concentration on elastic modulus remains important and often overlooked (Enemchukwu et al., 2016). In order to quantify the impact of RGD peptides on the mechanical properties of the gel we measured the shear modulus of gels of different concentration while also varying RGD molarity. This showed that the incorporation of 100 μM RGD peptides to the hydrogel network had a very limited impact on the gel stiffness. Increasing the RGD dose up to 1,200 μM led to a 32% decrease of elastic modulus in the stiffest gels (Figure 2B). This RGD-mediated decrease in stiffness is relatively modest when compared to the impact of varying PEG concentration. Altogether, the measured rheological data showed that varying PEG concentration allowed the production of soft (around 0.5 kPa), intermediate (2–4 kPa) and stiff (around 7 kPa) gels while the measured stiffness of the Matrigel scaffolds was several orders of magnitude lower. Furthermore, we also investigated the impact of gel proteolytic degradation by tuning the matrix metalloproteases sensitivity of PEG hydrogels according to Patterson and Hubbell (2010). There we found that cell survival was independent of the proteolytic degradation of the substrate (Figure S3). We also demonstrated that the most sensitive gels crosslinked with the 16R peptide allowed systematically for a higher efficiency of cyst formation across the entire range of gel stiffness and RGD concentrations (Figure S4).

When we quantified the average cyst size, we found that softer gels favored the formation of larger cysts with the largest cysts being systematically produced in Matrigel (approximatively 20% larger than in the best PEG condition). In addition, this experiment also showed that the presence of RGD, in a dose dependent manner, was critical for the formation of large cysts (Figure 2C). Interestingly, the measurement of cyst sizes across the nine RGD/Stiffness conditions showed that while increasing RGD concentration in soft gels dramatically increased cyst size, it only had a much lower (but still significant *p* < 0.001) effect in stiff gels. This indicates that stiffness seems to be the parameter that determines the possible maximum cyst size while the addition of RGD allows for the realization of that potential. Conversely, high stiffness clearly overrides the effect of integrin engagement and prohibits the formation of larger NRC cysts. This trend was further confirmed when we looked at the frequency of cyst formation (Figures 2D,E). There we found that increasing the elastic modulus of the hydrogels significantly reduced the frequency of cyst formation. When looking at maximal cyst formation efficiency we found that Matrigel and soft synthetic gels (1,200 μM RGD) had reached comparable levels. We also found that removing RGD from soft PEG drastically limited cyst formation (Figure S5). Finally, when quantifying cyst formation efficiency over 10 days we confirmed that both soft PEG and Matrigel imposed a similar logarithmic increase. Increasing the matrix stiffness was sufficient to significantly (*p* < 0.001) reduce this efficiency while the logarithmic trend was maintained (Figure 2E). Taken together these data clearly show that both, cholangiocytes cyst formation frequency and size are determined to a large extent by variation in the elastic modulus of the substrate. The presence of RGD ligand in high concentration further improved this trend.

### Establishment of Cyst Polarity

**Figure 3:**
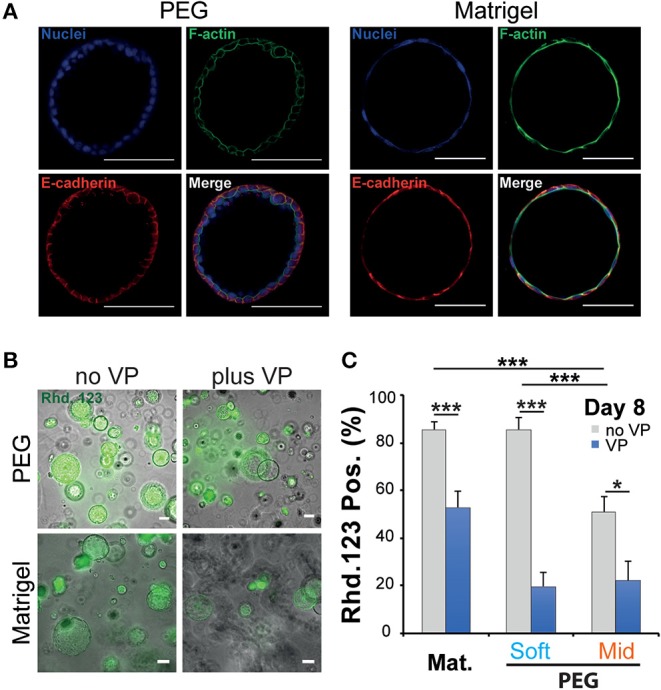
Phenotypic and functional assessment of Normal Rat Cholangiocytes (NRC) cysts produced in both polyethylene glycol and Matrigel hydrogels. **(A)** Scanning confocal microphotographs of immunostained NRC cysts after 10 days of culture. Both structures stain positive for actin (apically) and E-cadherin (basolaterally). **(B,C)** Widefield microphotographs and subsequent quantification showing the accumulation of rhodamine 123 (Rhd. 123) in the lumen of NRC cysts. Adding the channel blocking agent verapamil (VP) reduced this accumulation. * and *** Significant at *p* < 0.05 and *p* < 0.001 respectively. *n* = 4. Error bars represent SEM. Scale bar equal 100 μm.

When cultured in 3D, epithelial cells such as the kidney cell line (MDCK) self-assemble in hollow spheres and recapitulate the morphogenetic program leading to the production of rudimentary epithelial organoids (McAteer et al., 1986). Detailed analysis showed that interactions between key integrins and the extracellular matrix (ECM) regulate the establishment of cell polarity and morphogenesis (Manninen, 2015). In both cases whether NRC cysts can become polarized upon cultured in a synthetic 3D scaffold was investigated first by immunochemistry and rhodamine transport assay. There we found that large NRC cysts stain positive for basolateral E-Cadherin and apical F-actin (Figure 3A; Figure S6). Canonical epithelial polarity was observed in cysts of different dimensions across the tested 3D culture conditions. NRC cysts with inverted polarity could also be observed as described elsewhere (Enemchukwu et al., 2016). However, these observations were very seldom and did appear to correlate with either stiffness or RGD concentration. When looking at the shape of the nuclei of the cholangiocytes lining the cyst wall, we found that in Matrigel these structures seemed to be more elongated and placed tangentially to the formed circle whereas in PEG the nuclei seemed rounder, organized into a mixed mono- and/or double-layer structure (Figure 3A). *In vivo*, the morphology of the nuclei in the lining of the bile duct seems to vary from round to oval with preferentially a basal localization in the cell. On the functional level we found that, in MT or in PEG hydrogels, formed cysts could accumulate rhodamine 123 in their lumen and that this accumulation was blocked by the addition of verapamil in the culture medium. This clearly indicates that the accumulation of rhodamine is resulting from transmembrane channel protein-multidrug resistance protein-1 (MDR1) activity and not passive diffusion (Figure 3B). Furthermore, we could show that the frequency of rhodamine accumulating cysts was reduced when matrix stiffness was increased (Figure 3C). These data suggest that NRC cysts cultured in either PEG or MT are showing apicobasal polarity and functioning as mature epithelial cysts.

### Morphogenesis and Lumen Formation

**Figure 4:**
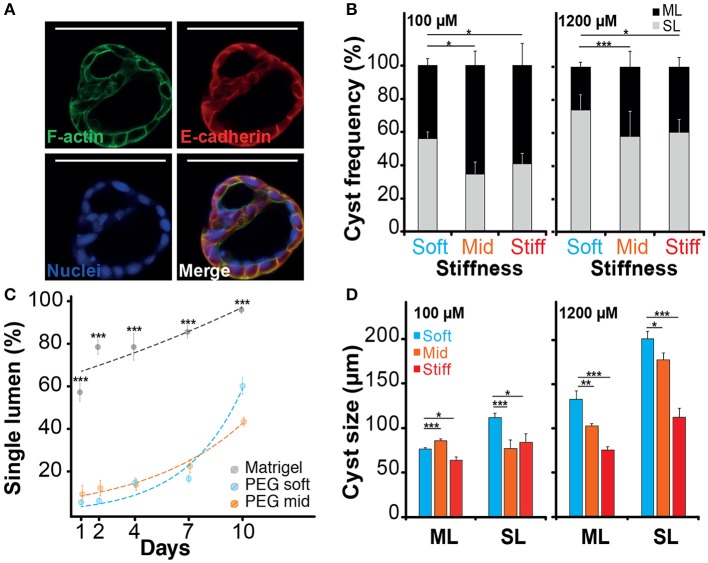
Impact of the microenvironment on lumen formation. **(A)** Immunostaining of f-actin and E-cadherin localization in normal rat cholangiocyte (NRC) cyst with multi-lumen phenotype. The observation of Normal Rat Cholangiocytes (NRC) cysts formation in different matrices showed that some NRC cysts could comprise multiple lumens. **(B)** Quantification of single lumen formation of NRC cysts during 10 day. The quantifications showed that this phenomenon was prevalent in PEG hydrogel and marginal in Matrigel. We also found that the fraction of single lumen cysts in PEG tended to exponentially increase over time. **(C)** The analysis of the “multi-lumen” and “single-lumen” phenotype frequency indicated that a soft microenvironment containing 1,200 μM RGD caused the lowest rates of multi-lumen formation. **(D)** Finally, cyst size analysis showed that variation in RGD concentration seemed to alter the multi-lumen/single-lumen ratio. SL and ML is for single-lumen and multi-lumen, respectively. *, **, and *** Significant at *p* < 0.05, *p* < 0.01, and *p* < 0.001, respectively. *n* = 4. Error bars represent SEM. Scale bar equal 100 μm.

When imaging the different structures obtained after culturing NRC in PEG and MT hydrogels, we have identified different cell organizations including large cysts including a unique lumen bordered by a thin layer of cells (SL) (Figure 3A; Video S1), spherical cell structures containing multiple gaps (ML) (Figure 4A; Figure S7) and small and compact cell aggregates. We found that the hydrogel recipe was determining to a certain extent the frequency of each structure. Quantifications preformed on different scaffolding conditions showed that decreasing the hydrogel stiffness and increasing the RGD concentration increased the frequency of SL structures (Figures 4B–D). More interestingly the effect of stiffness seemed to plateau for gels between 2.5 and 4.5% (w/v) PEG. We observed that NRCs cultured in 2.5% PEG hydrogel (G′ ≈ 0.5 kPa) showed a SL frequency of 73 and 56% for high and low RGD concentrations respectively. The formation of SL was decreased by 20% when using stiffer gels. An even further decrease was observed when using low RGD in the stiffest gels. This indicates that above a certain elasticity threshold (between 0.5 and 2.5 kPa) the luminal organization seems to be rather independent of the hydrogel stiffness whereas RGD density remains an important determinant for the formation of single lumen with normal polarity. Although comparable at day 10, the frequency of SL cyst formation did not appear to follow identical trajectories in PEG vs. MT. Observing the dynamics of SL cyst formation over the course of 10 days revealed that the formation of SL cysts seemed to follow an exponential law in PEG whereas cyst formation dynamics was found more linear in MT. These dynamics reveal that the gap in percent of SL cyst formation is strongly decreasing over time when comparing MT and PEG (Figure 4C). When investigating cyst organization in relation to size we found that ML cysts are significantly smaller than SL cysts. This observation remained true for longer observations (2 weeks plus) (Figure S7). We also found that limiting the dose of RGD clearly restricted the growth of the produced ML cysts (Figure 4D). This experiment also showed that cyst size and organization are not fully correlated and that the two parameters could respond to different microenvironment cues.

## Discussion

The ECM-cholangiocyte interaction is essential for the formation of the biliary tree. *In vitro*, the ECM is classically mimicked by natural hydrogels known to promote well-cholangiocyte differentiation and morphogenesis (Tanimizu et al., 2007). Understanding the role of the microenvironment cues on bile duct formation is clearly limited by the type of assays that can recapitulate this process *in vitro*. On one hand, hydrogels based on naturel ECM allow to faithfully recapitulate key morphogenetic processes (Tanimizu et al., 2007). However, mechanistic studies in these systems are difficult because of the intertwined biochemical and biophysical signaling. On the other hand, synthetic hydrogels are more amenable to experiment design and systematic variation (Ranga et al., 2014) but less efficient in the support of biological processes. In this work we clearly benefitted from these developments in order to produce synthetic, and fully defined scaffolds capable of efficiently supporting cholangiocyte cyst formation.

Structure wise, epithelial organoids are organized as a monolayer of polarized epithelial cells surrounding a central lumen. Two typical structures can emerge from this basic concept: cysts or tubes. Nevertheless, the precise underlying morphogenetic mechanism remains elusive and the contribution of the microenvironment is yet to be quantified (Yu et al., 2007; Martin-Belmonte et al., 2008). Other studies have shown that primary biliary mouse epithelial cells (BECs) do form cysts from a single cell when cultured in Matrigel and that the resulting cyst formation efficiency is determined by the initial cell number in the aggregates (Rizki-Safitri et al., 2018). Similarly, the encapsulation of single MDCK cells was found sufficient to trigger cyst formation in synthetic and natural hydrogels (Martin-Belmonte et al., 2008; Enemchukwu et al., 2016). Interestingly, the cell aggregation route for the formation of MDCK cysts was also shown pertinent when these cells were encapsulated at high density. Unlike NRCs or MDCKs, other cell types showing very limited proliferation capabilities such as epithelial lung cells (AT II) still produce cysts with sizes directly linked to initial cell density (Yu et al., 2007). Our experiments showed that the encapsulation of aggregates of multiple NRC resulted in cyst formation by cellular rearrangement whereas single cells systematically failed to proliferate and organize. Similar findings were made for NRC cyst formation in MT with the difference that single cells could migrate and aggregate before proliferating and forming cysts.

When stiffness was considered, we demonstrated that matrix elasticity variation modulates cyst formation (Figure 2D). Strikingly, cyst formation was found to be very comparable in soft PEG gels and in MT despite the order of magnitude difference in elastic moduli. This is probably due to the fact that both MT and soft PEG conditions are still below the elastic modulus of a healthy liver (~1 kPa) (Yeh et al., 2002). Globally, the cyst formation process was found to tolerate departure from the healthy liver condition. Even recapitulating fibrotic liver conditions (~5 kPa, Stiff PEG) did not fully block the process. Very interestingly this observation is consistent with the fact that an abnormal increase in intrahepatic biliary mass is taking place during the earliest stages of liver fibrogenesis (Schuppan et al., 2018). In comparison, when encapsulating MDCK cells cyst formation is completely blocked at 7 kPa (Enemchukwu et al., 2016). Matrix elasticity was found to play a key role in other systems including intestinal organoids and lung adenocarcinoma spheres (Gill et al., 2012; Raza et al., 2013; Gjorevski et al., 2016; Broguiere et al., 2018). In these systems, relatively high initial stiffness was needed to drive cell compaction. A quick softening over time was also important in order to help with the 3D rearrangement of cells.

When considering the biochemical cues, we first tried to recapitulate physiologically relevant integrin ligand densities by adjusting the RGD peptide concentration in the PEG hydrogels. However, as the *in vivo* density of RGD motifs accessible to intrahepatic cholangiocytes is hard to assess we employed 100 μM as the best approximation based on the fibrin structure (Raeber et al., 2005) and 1,200 μM as the most efficient concentration for cysts derivation in an equivalent MDCK model (Enemchukwu et al., 2016). In these conditions NRC cyst formation frequency was found independent of the level of integrin engagement. This is in good agreement with the work performed on MDCK cells in synthetic hydrogels (Enemchukwu et al., 2016). This way we found that cyst size (growth of cysts) and thus lumen formation relied on integrin engagement (Figures 2C, 4B). The growth in diameter of NRC cysts is known to be fueled by fluid secretion into the cyst lumen and cell proliferation (Neufeld et al., 1992; Doctor et al., 2007). Our results suggest that hydrogel elasticity and cell adhesion can synergistically determine the growth of NRC cysts. When comparing Matrigel and PEG-based systems, Matrigel systematically produced larger cysts. This might be due to the different mechanisms of lumen formation, where NRC cysts cultured in MT achieved cyst polarization at an early time point of cyst formation followed by rapid cyst expansion. The formation of a central lumen is a key feature of epithelial morphogenesis. The mechanism of lumen formation in MDCK cysts grown in natural hydrogels depends on the used ECM composition. MDCK cells cultured in collagen I showed later cell polarization during cyst formation and formed lumen by center cell apoptosis passing through a multi lumen state, whereas MDCK cysts grown in laminin rich Matrigel induced early cell polarization and formed lumen through cell proliferation with almost negligible center cell apoptosis (Martin-Belmonte et al., 2008). This was further confirmed when MDCK cysts were found to form luminal space even when apoptosis was suppressed, hinting to the involvement of multiple lumen forming mechanisms (O'Brien et al., 2002). In comparison, cysts derived from lung epithelial cells cultured in natural hydrogels formed lumen through cell rearrangement without cell proliferation and cell apoptosis (Yu et al., 2007).

In general, epithelial cysts grown in natural hydrogels clearly show single lumen phenotype predominantly. In synthetic hydrogels different phenotypes were observed for NRC and MDCK cells (Enemchukwu et al., 2016). We demonstrated that NRC cyst grown in PEG hydrogels or in MT both formed lumen with a typical apical-basolateral polarity (Figure 3A). The accumulation of Rhodamine 123 in the produced cysts hints to a proper and functional organization of the cells. Staining for primary cilia and quantifying the response to farnesoid X receptor could help further confirm this aspect. The mechanisms of lumen formation for cysts cultured in PEG hydrogels was found to be based on cell rearrangement, rather than on early cell polarization as shown in MT. Hydrogel stiffness and RGD concentrations were found to be linked to polarization and to the frequency of single lumen formation. A high integrin engagement was shown to enhance the single lumen phenotype over multi-lumen phenotype in NRC cysts for Soft and Stiff PEG hydrogels, respectively. A positive correlation between lumen formation and adhesive ligand density of a synthetic scaffold was also demonstrated in lung adenocarcinoma models (Gill et al., 2012). Not providing polarization cues in synthetic 3D culture systems systematically delays the acquisition of the cyst polarity (Martin-Belmonte et al., 2008; deLeon et al., 2012). For MDCKs a delayed polarization led to the production of multiple lumens that could fuse at later stages. The size of multi-lumen MDCK cysts cultured in natural and synthetic hydrogels was reported to be similar to cysts containing a single lumen (Martin-Belmonte et al., 2008; Enemchukwu et al., 2016). In our hands a majority of NRC cysts with a single lumen derive from multi-lumen structures. In some cases, these multi-lumen cysts persist. In the MDCK system, the multi-lumen cysts phenotype was found associated with the mislocalization of basal components leading to the accumulation of ECM in the luminal space (Torkko et al., 2008). Unlike MDCKs, the NRC multi-lumen cysts were found to be systematically smaller when compared to single-lumen cysts. The suppression of outer layer coat complex II in human intestinal epithelial cells (Caco-2) cultured in 3D resulted in cysts with defective lumen expansion attributed to a faulty ECM signaling when spatially directing cell division. Similar to multi-lumen NRCs, these cysts show a reduced cell number, multi-lumen formation, and smaller sizes (Townley et al., 2012). We speculate that providing NRCs cultured in PEG with a stronger polarization cues by replacing RGD with laminin or laminin derived peptides could significantly stimulate single-lumen formation and expansion of NRC cysts.

In summary, we have established a tunable PEG-based hydrogel system that can be used for cholangiocyte cyst formation and morphogenesis studies. We demonstrated that making these gel cell responsive by adding MMP sensitivity and integrin ligand contributed to meet the cyst production capacity of NRCs encapsulated in MT. We expect that in the future, this system will be employed to study pathogenicity of biliary diseases and to identify therapeutic targets.

## Data Availability Statement

The datasets generated for this study are available on request to the corresponding author.

## Author Contributions

AF carried out the experiments performed in PEG hydrogel including cell culture, hydrogel casting, and cell encapsulation, helped with the main figures, and arranged the supplementary figures. NM Performed the rheology tests on PEG gels. LB and EG carried out the experiments in natural hydrogels, including cell culture, cell encapsulation, microscopy, and rheology tests on Matrigel. SG, PD-W, and AF produced together a first outline of the manuscript. PD-W wrote part of the introduction. SG arranged the four main figures, wrote the abstract, part of the introduction, the results, and the discussion. All the authors reviewed the manuscript.

### Conflict of Interest

The authors declare that the research was conducted in the absence of any commercial or financial relationships that could be construed as a potential conflict of interest.
