# Supplementary material for: Biophysical Control of Bile Duct Epithelial Morphogenesis in Natural and Synthetic Scaffolds
Source: Front Bioeng Biotechnol. 2019 Dec 13;7:417. doi: 10.3389/fbioe.2019.00417 (PMC6923240; doi:10.3389/fbioe.2019.00417)
Supplement: Supplementary file 3 [file Data_Sheet_1.docx]

***Supplementary Material***

Biophysical Control of Bile Duct Epithelial Morphogenesis in natural and synthetic scaffolds.

Anette Funfak^1^, Latifa Bouzhir^2^, Emilie Gontran^2^, Nicolas Minier^1,3^, Pascale Dupuis-Williams^2,4,*^, Samy Gobaa^1,*^

^1^ Institut Pasteur, Biomaterials and Microfluidics core facility, C2RT, Paris, France

^2^ UMR-S1174 Institut National de la Santé et de la Recherche Médicale, Université Paris-Sud, Orsay, France.

^3^ Université de Technologie de Compiègne, Alliance Sorbonne Université, Compiègne, France

^4^ École Supérieure de Physique et de Chimie Industrielles de la Ville de Paris, Paris, France.

* These authors contributed equally to the work.

Correspondence

Prof. Pascale Dupuis-Williams

[pascale.dupuis-williams@u-psud.fr](mailto:pascale.dupuis-williams@u-psud.fr)

Dr. Samy Gobaa

[samy.gobaa@pasteur.fr](mailto:samy.gobaa@pasteur.fr)

**Movie S1.** Three-dimensional nucleus location of NRC cells within different 10-day old cysts cultured in 4.5% PEG-VS hydrogels supplemented with 1200µM RGD. Scale bar:100µm

**Movie S2.** NRC cysts growth filmed over 48h in a PEG-VS hydrogel with intermediate stiffness containing 1200µM RGD.


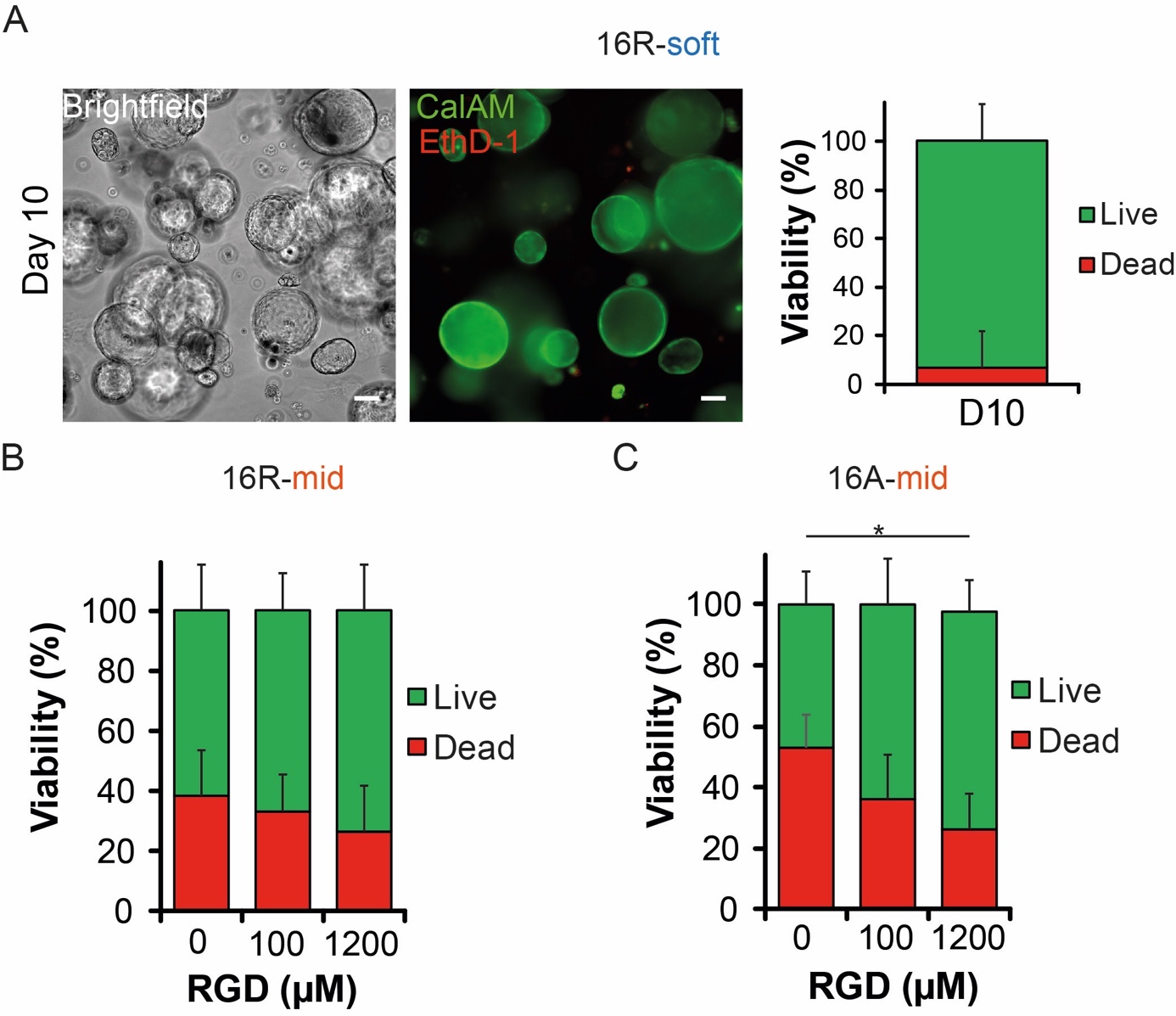


**Figure S3.** NRC cyst viability at Day 10 for PEG-VS hydrogels crosslinked with a fast (16R) and slow (16A) MMP-degradable peptide sequence. A) Representative brightfield and z-stack confocal images of 10-d-old NRC cysts cultured in a soft PEG-VS hydrogel crosslinked with a fast degradable crosslinker sequence and functionalized with 1200µM RGD (left) and percentage of cell viability at day 10 assessed by CalAM (Live) and EthD-1 (Dead) staining (right). B) Percentage of cell viability for different RGD ligand concentration assessed by CalAM (Live) and EthD-1 (Dead) staining of 10-d-old NRC cysts cultured in 4.5% PEG-VS hydrogel crosslinked with a fast MMP-degradable peptide. C) Percentage of cell viability for different RGD ligand concentration assessed by CalAM (Live) and EthD-1 (Dead) staining of 10-day old NRC cysts cultured in 4.5% PEG-VS hydrogel crosslinked with a slow MMP-degradable peptide. * significant at p<0.05. Error bars represent SD. Scale bar: 100µm.


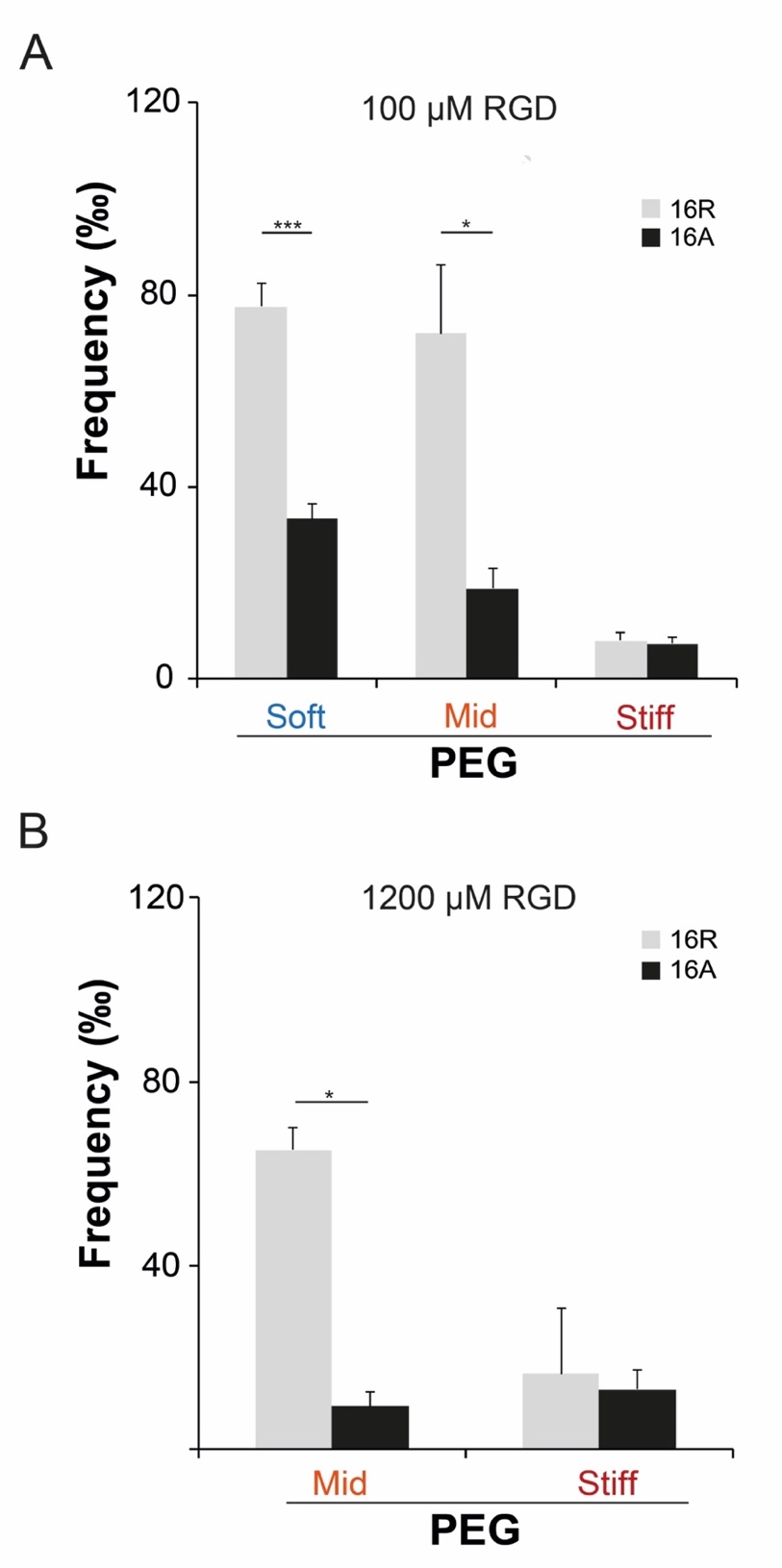


**Figure S4.** NRC cyst formation. (A) Comparison of cyst formation efficiency between fast and slow MMP-degradable hydrogels with soft, intermediate and stiff matrix when containing 100µM RGD. (B) Comparison of cyst formation efficiency between fast (16R) and slow MMP-degradable (16A) hydrogels with intermediate and stiff matrix when containing 1200µM RGD. *, **, *** significant at p<0.05, p<0.01 and p<0.001 respectively. Error bars represent SEM.


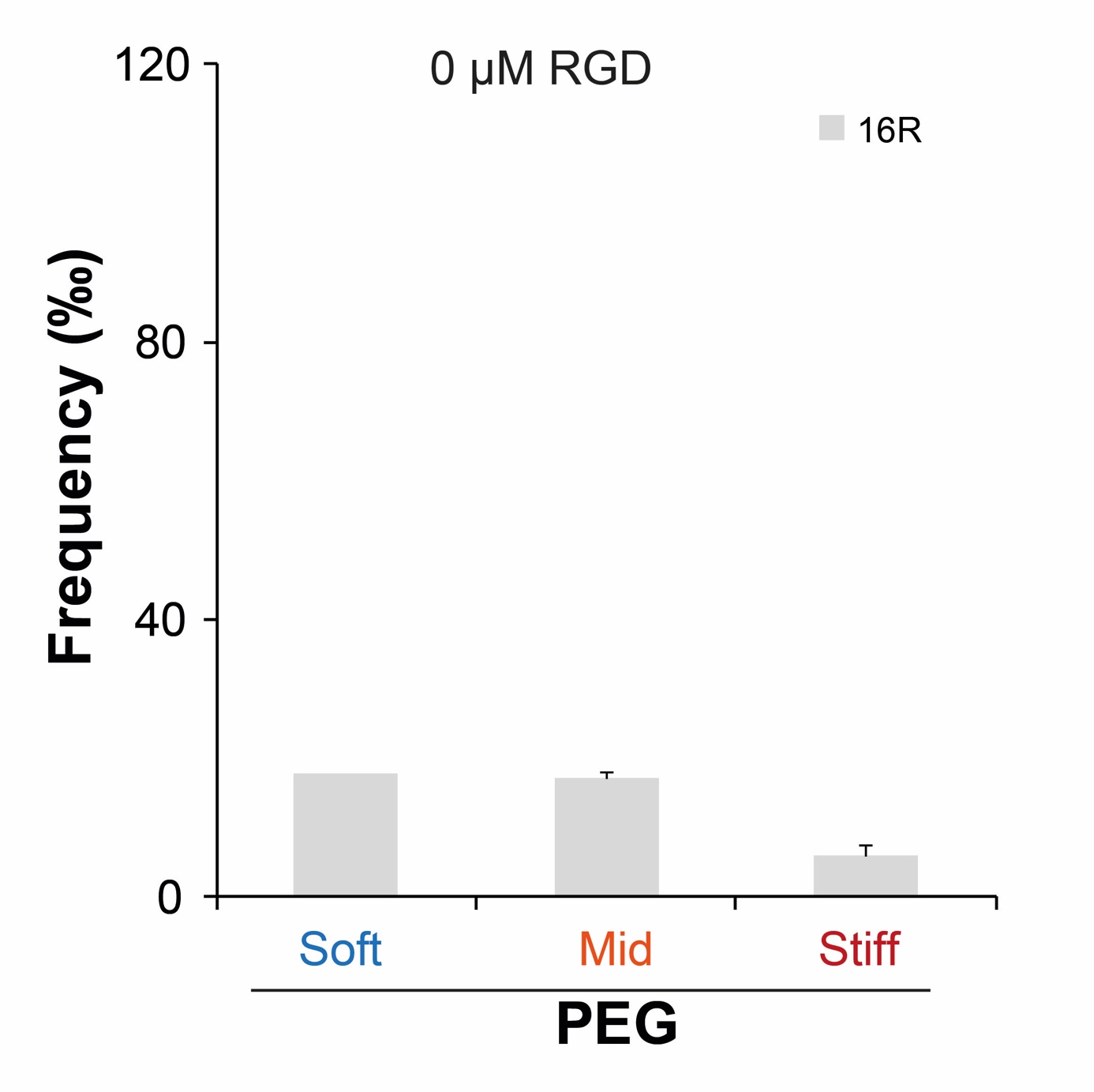


**Figure S5.** NRC cyst formation. Number of cysts formed in soft, intermediate and stiff hydrogels in absence of the RGD ligand. Error bars represent SEM.


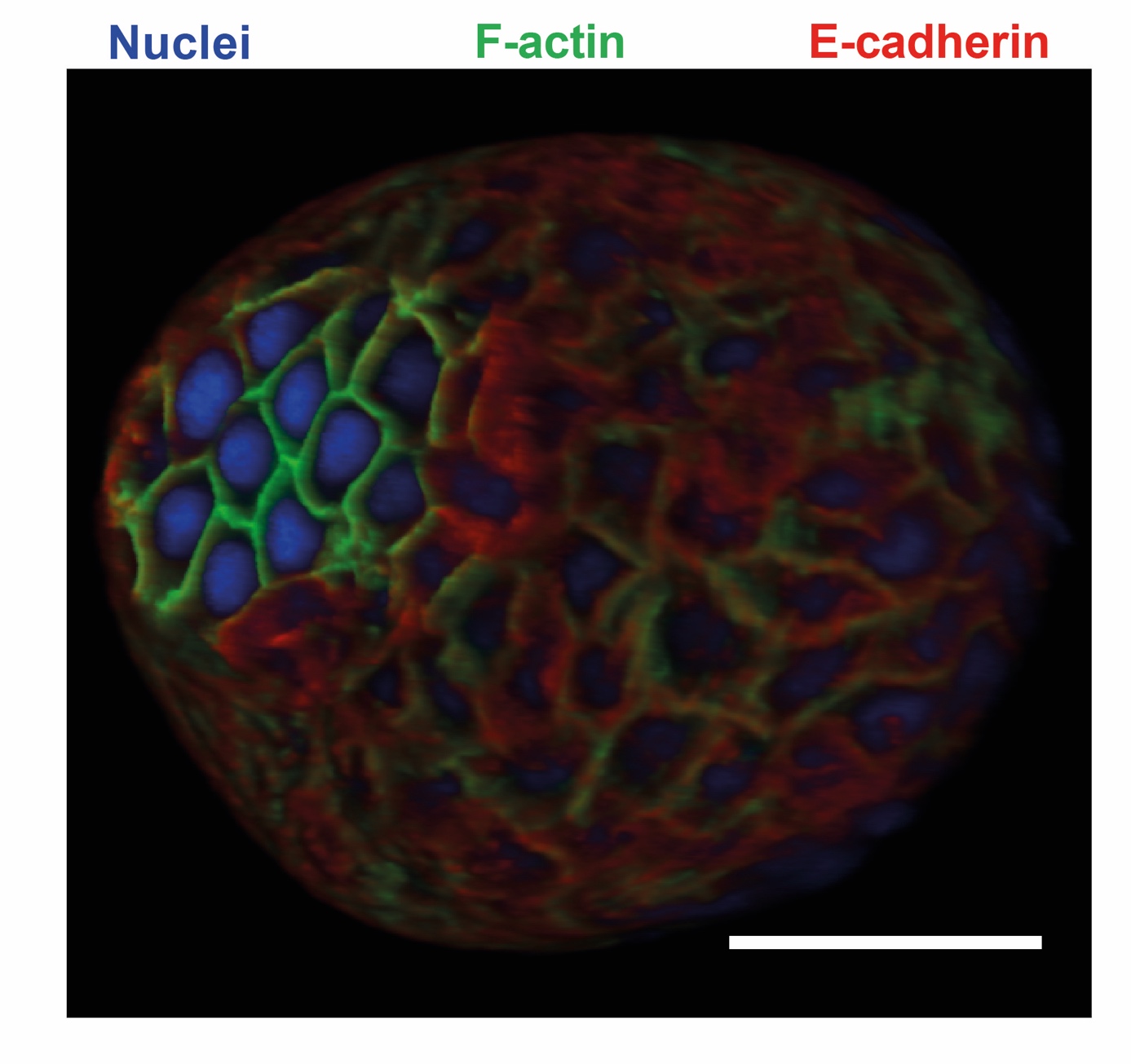


**Figure S6.** NRC cyst cultured in PEG-VS hydrogels express apical-basolateral polarization. 3D immunostaining image of a cysts marked for basolateral marker E-cadherin and apical marker f-actin. Scale bar: 100µm.

**
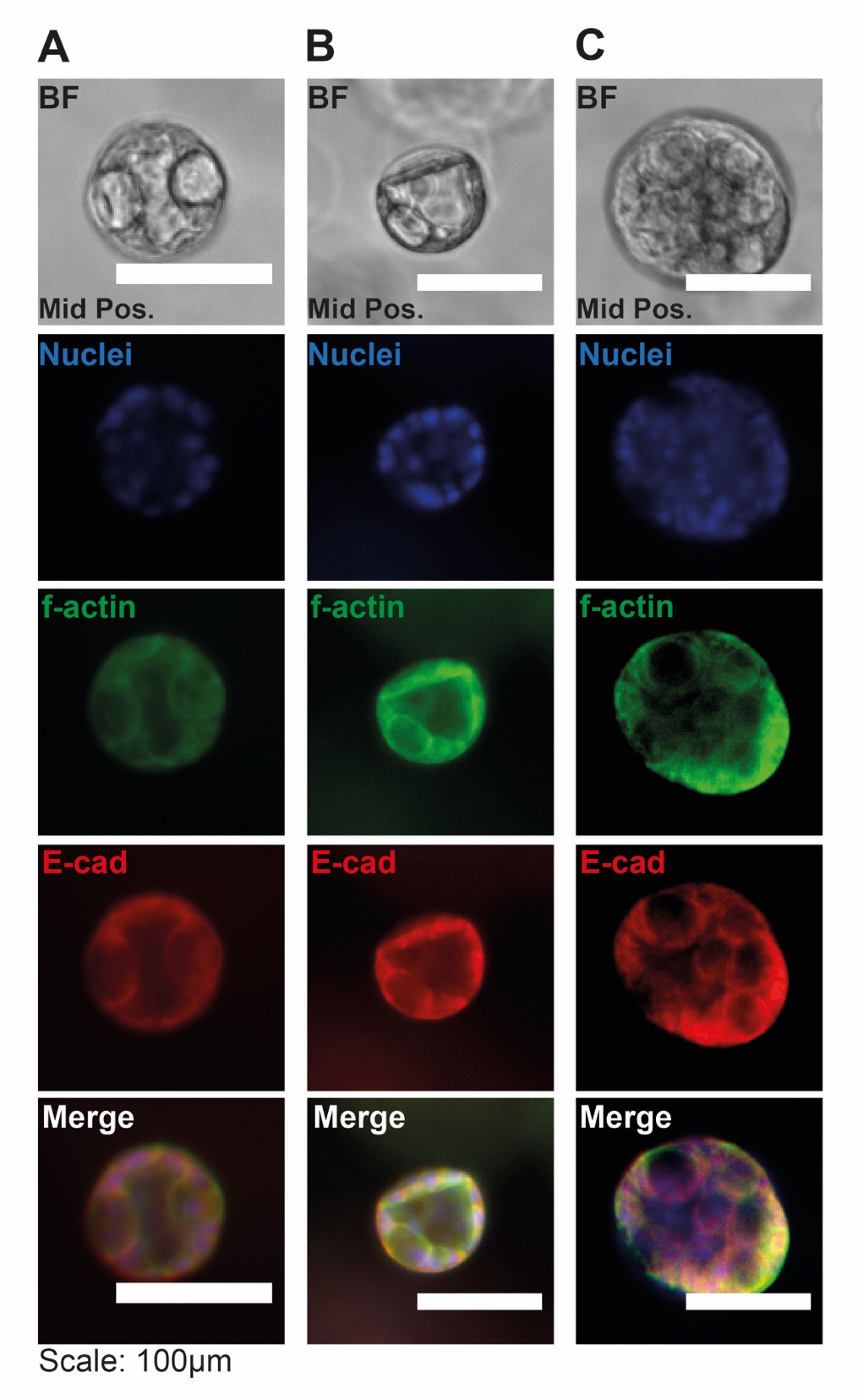
**

**Figure S7.** Normal Rat Cholangiocyte (NRC) cysts can retain a multi-lumen phenotype over 18 days of culture in 4.5% PEG-VS hydrogels containing 1200 µM RGD. (A) Three-lumen cyst, (B) two-lumen cyst and (C) 8+ lumen cyst. BF: Brightfield. E-cad: E-cadherin. Scale bar: 100µm.
